# Planning protected areas network that are relevant today and under future climate change is possible: the case of Atlantic Forest endemic birds

**DOI:** 10.7717/peerj.4689

**Published:** 2018-05-24

**Authors:** Mariana M. Vale, Thiago V. Souza, Maria Alice S. Alves, Renato Crouzeilles

**Affiliations:** 1 Departamento de Ecologia, Universidade Federal do Rio de Janeiro, Rio de Janeiro, Brazil; 2 Instituto de Recursos Naturales, Laboratorio Internacional de Cambio Global, Madrid, Spain; 3 Brazilian Research Network on Global Climate Change—Rede Clima, São José dos Campos, São Paulo, Brazil; 4 Programa de Pós-Graduação em Ecologia, Universidade Federal do Rio de Janeiro, Rio de Janeiro, Brazil; 5 Ecology Department, Universidade do Estado do Rio de Janeiro, Rio de Janeiro, Brazil; 6 Rio Conservation and Sustainability Science Centre, Pontifícia Universidade Católica do Rio de Janeiro, Rio de Janeiro, Brazil; 7 Instituto Internacional de Sustentabilidade, Rio de Janeiro, Brazil

**Keywords:** Biodiversity, Decision-making, GIS, Brazil, Systematic conservation planning, Ecological niche modelling

## Abstract

**Background:**

A key strategy in biodiversity conservation is the establishment of protected areas. In the future, however, the redistribution of species in response to ongoing climate change is likely to affect species’ representativeness in those areas. Here we quantify the effectiveness of planning protected areas network to represent 151 birds endemic to the Brazilian Atlantic Forest hotspot, under current and future climate change conditions for 2050.

**Methods:**

We combined environmental niche modeling and systematic conservation planning using both a county and a regional level planning strategy. We recognized the conflict between biodiversity conservation and economic development, including socio-economic targets (as opposed to biological only) and using planning units that are meaningful for policy-makers.

**Results:**

We estimated an average contraction of 29,500 km^2^ in environmentally suitable areas for birds, representing 52% of currently suitable areas. Still, the most cost-effective solution represented almost all target species, requiring only ca. 10% of the Atlantic Forest counties to achieve that representativeness, independent of strategy. More than 50% of these counties were selected both in the current and future planned networks, representing >83% of the species.

**Discussion:**

Our results indicate that: (i) planning protected areas network currently can be useful to represent species under climate change; (ii) the overlapped planning units in the best solution for both current and future conditions can be considered as “no regret” areas; (iii) priority counties are spread throughout the biome, providing specific guidance wherever the possibility of creating protected area arises; and (iv) decisions can occur at different administrative spheres (Federal, State or County) as we found quite similar numerical solutions using either county or regional level strategies.

## Introduction

Ongoing climate change has already affected different species in every ocean and continent worldwide (Walther et al., 2002; Parmesan & Yohe, 2003; Root et al., 2003). Redistribution of species’ range in response to climate change are particularly common (e.g., Parmesan et al., 1999; Perry et al., 2005; Chen et al., 2011; Lenoir & Svenning, 2015), and predictions of widespread range shifts and contraction in the near future are particularly worrisome (Pecl et al., 2017). The establishment of protected areas remains a key strategy in biodiversity conservation (Crouzeilles et al., 2013a). Approximately 13% of the global land surface is under protected areas (Jenkins & Joppa, 2009). In the future, however, species’ redistribution due to climate change is likely to affect species’ representativeness and persistence in current protected area networks (Hannah et al., 2007; Araújo et al., 2011; Loyola et al., 2013; Lemes, Melo & Loyola, 2014).

To minimize the negative impacts of climate change on biodiversity, protected areas networks must be planned considering not only current species’ distribution, but also its response to climate change (Loyola et al., 2013; Hannah et al., 2007). An obvious solution for this problem is combining ecological niche modeling and systematic conservation planning in light of climate change scenarios. However, few studies have combined both approaches to propose effective protected areas network under climate change (Hannah et al., 2007). Notable exceptions are a study on plants in Europe (Araújo et al., 2004), a multi-taxa study in Mexico, South Africa, and Western Europe (Hannah et al., 2007), and an amphibian study in Brazil (Loyola et al., 2013). These studies have all focused on biological targets in protected area planning, finding a varying degree of species’ loss from selected reserves under future climatic conditions. To make cost-effective decisions, however, studies must recognize and take into account the conflict between biodiversity conservation and socio-economic demands (Pinto & Grelle, 2011; Crouzeilles et al., 2015). In areas that have similarly high biodiversity, the ones that are less economically attractive or densely populated should be prioritized for protection, minimizing conflict and increasing protected areas’ likelihood of success in achieving its conservation goals.

Several studies have used human population density as a surrogate for socio-economic development when planning protected areas network in Africa (Balmford et al., 2001), Europe (Araújo, Williams & Turner, 2002), Australia and North America (Luck et al., 2004), and Brazil (Diniz-Filho et al., 2006; Pinto et al., 2007; Pinto & Grelle, 2011). The use of human population density is based on the following assumptions: (i) densely populated areas experience a higher land use conflict between biodiversity conservation and human development (Balmford et al., 2001; Luck, 2007; Pinto & Grelle, 2011); (ii) is a strong predictor of species extinction risk (e.g., Cardillo et al., 2005); (iii) is directly related to habitat conversion (Myers et al., 2000; Laurance et al., 2002); and (iv) is a proxy for opportunity cost (Luck et al., 2004; Rangel et al., 2007).

In order for planned protected areas network to remain effective under climate change conditions, it is paramount to minimizing conflict between conservation and development at the level at which top-down decision-making occurs (e.g., at the federal, state or county administrative spheres). This kind of approach is timely for the Brazilian Atlantic Forest, one of the five hottest hotspots in the world, i.e., areas with exceptionally high biodiversity and that have lost >85% of its original forest cover (Laurance, 2009). Despite its conservation relevance, knowledge of the possible impacts of climate change on Atlantic Forest biodiversity is still scarce (Vale, Alves & Lorini, 2009). The few existing studies predict range contraction for most species in the future (e.g., Souza et al., 2011; Loyola et al., 2013; Lemes, Melo & Loyola, 2014; Loyola et al., 2014). The Atlantic Forest hosts 70% of the Brazilian population and 80% of its gross domestic product (MMA, 2013). It is not surprising, therefore, that only ca.12% of its original 1,500 km^2^ forest cover remains, 9% of which under protected areas (Ribeiro et al., 2009).

Here we quantify the effectiveness of planning protected areas network in the Brazilian Atlantic Forest, both currently and under future climate change conditions. We selected the most cost-effective areas to establish protected areas network, focusing on maximizing conservation of 151 endemic bird species, while minimizing co-occurrence with densely populated areas at different scales (county versus regional level strategy). Our key question is whether planning protected areas network under current conditions is similar as planning it under future climate conditions.

## Materials & Methods

We combined two methodological approaches, ecological niche modeling and systematic conservation planning, to identify the most cost-effective areas to establish protected areas network in under current and future climatic conditions. Ecological niche models were based on five algorithms under a business-as-usual climate change scenario. We combined algorithms in a final binary ensemble model and then clipped to the Atlantic Forest remnants, excluding areas where forest no longer exists. We maximized the representation and persistence of each endemic bird species considering different conservation targets and minimized co-occurrence with densely populated areas, using counties as the unit of analysis. We also simulated the impact of more clumped solutions. Finally, we contrasted the most cost-effective solution in under each climatic condition, current and future, using both county and regional level strategy (i.e., simulating more clumped solutions).

### Study area

The Brazilian Atlantic Forest stretches along the coast of Brazil, embracing a large latitudinal and altitudinal gradient that creates a number of different biogeographical sub-regions within the biome (Silva, Sousa & Castelletti, 2004; Ribeiro et al., 2009). We used the geographic boundaries of the Atlantic Forest established under the Brazilian legislation (Law No. 11.428 from 22 December 2006). Our analysis included the 2,688 counties with ≥75% of its territory within the limits of the Atlantic Forest biome. Biome and county spatial data were downloaded from the Brazilian Institute of Geography and Statistics (http://downloads.ibge.gov.br/downloads_geociencias.htm).

### Ecological niche modeling

We updated Bencke et al. (2006)’s list of Atlantic Forest endemic bird species. We used the digital maps of BirdLife International (BirdLife International and NatureServe 2006, available at http://www.iucnredlist.org/technical-documents/spatial-data), and elaborated a list of species that have at least 80% of their distribution within the original extent of the Atlantic Forest biome, as defined by the ecoregions (Olson et al., 2001, available at http://www.worldwildlife.org/publications/terrestrial-ecoregions-of-the-world). We than examined the occurrence records provided by WikiAves (http://www.wikiaves.com.br/), a citizen science project that compiles bird records, with associated photos or song recordings, and kept only the species that had a clear concentration of records within the Atlantic Forest. We compiled occurrence records using original data, the literature and major ornithological collections in the region (Rio de Janeiro National Museum in Rio de Janeiro State, University of São Paulo Museum in São Paulo State, Professor Melo Leitão Bilogy Museum in Espírito Santo State, Capão da Imbuia National Museum in Paraná State, and João Moogen Zoology Museum at Minas Gerais State). We looked at every occurrence record, excluding those with will-defined geographic coordinates or that clearly fell outside species range as defined by the scientific literature, the digital maps of BirdLife International, or the range description provided the IUCN Red List of Threatened Species. We also used the IUCN Red List to exclude species extinct in the wild. We excluded species with fewer than 12 reliable occurrences. At the end, we had 151 bird species and 5,482 unique occurrence records (see Table S1 species’ list and number of occurrence records).

We used bioclimatic variables (Hijmans et al., 2005) as predictors in the ecological niche modelling. We selected six variables that show relatively low correlation among each other in the Atlantic Forest (see Souza et al., 2011): (i) temperature seasonality, (ii) precipitation seasonality, (iii) maximum temperature of the warmest month, (iv) minimum temperature of the coldest month, (v) precipitation of the wettest month, and (vi) precipitation of the driest month. The data had 2.5 arc-minute spatial resolution, which is compatible with the spatial error associated with occurrence record’s location. Data under current conditions were downloaded from WorldClim (http://www.worldclim.org) and data for year 2050 were downloaded from the International Center for Tropical Agriculture (http://ccafs-climate.org) under the A2a business as usual greenhouse gas emission scenario (Nakicenovic & Swartz, 2000) according to the HadCM3 global circulation model, which has good performance and predictability for South America (Yin et al., 2013).

Ecological niche models were built in the ModEco software (Guo & Liu, 2010), using five modeling algorithms, which are complementary in terms of mathematical structure and input data requirements. We included two environmental envelope algorithms that use presence-only data (environmental envelope–BIOCLIM and environmental distance –DOMAIN), two statistical algorithms that use presence versus absence data (SVM and logistic regression –GLM) and one machine learning algorithms that uses presence versus background data (MAXENT). We used background points randomly distributed in the extent of analysis (ModEco’ default) totaling five times the number of species’ occurrence records used to calibrate the model. The algorithms also differed in the output format: BIOCLIM and DOMAIN generate binary projections (suitable versus unsuitable), while the remaining algorithms generate continuous projections of environmental suitability. For the latter, we used a threshold value, maximizing the number of occurrence records under suitable areas (sensitivity) and pseudo-absences under unsuitable areas (specificity). This threshold method produces final models with higher performance measures, and is not affected by the type of input data use, being suitable for all algorithms used (Liu, White & Newell, 2013).

We evaluated model’s performance through True Skill Statistics (TSS) on model’s binary outputs (Allouche, Tsoar & Kadmon, 2006) based on a confusion matrix built with true presence and pseudo-absence data. TSS values vary between −1 and +1, where negative values indicate very poor performance and a value of 1 indicates a perfect fit. For presence data we randomly selected 25% of species’ occurrence records. For pseudo-absence we randomly selected points outside species’ distribution, using IUCN /BirdLife International distribution maps (http://datazone.birdlife.org/species/requestdis). Following Lobo & Tognelli (2011), the number of pseudo-absences was 100 times that of presences. Models with TSS <0.4 were discarded.

We combined algorithms in a final binary ensemble model using the majority rule (Thuiller, 2004; Araújo & New, 2007). Given that we used five algorithms, we only selected areas as “environmentally suitable” if they were selected by at least three algorithms. Ensemble models were then clipped to the Atlantic Forest remnants, excluding areas where forest no longer exists. This procedure produced the final maps of “environmentally suitable areas” for each the species, under current and future climatic conditions, used in the systematic conservation planning analysis (see below). Therefore, environmentally suitable areas have both adequate climate and adequate forest cover for species’ occurrence, assuming that in the future the forest cover will remain nearly the same. We did not use a prediction of forest configuration in 2050 because: (i) there is not one available at a meaningful spatial resolution (e.g., the Land-Use Harmonization Project has a resolution of 50 km used by Zwiener et al., 2017, excludes at least 80% Atlantic Forest remnants according to Ribeiro et al., 2009), (ii) the Atlantic Forest Law (Law No. 11.428 from 22 December 2006) does not allow any further deforestation in the biome, and (iii) there is no prediction of a contraction of the Atlantic Forest due to climate change, as is the case for the Amazon (Salazar, Nobre & Oyama, 2007). The data on Atlantic Forest remnants was based on the map of Fundação SOS Mata Atlântica (SOS Mata Atlântica and INPE, 2012), derived from TM/Landsat 5, ETM/Landsat 7 or CCD/CBERS-2 images, available at a scale of 1:50,000 in vector format and depicting remnants ≥3 ha.

### Planning protected areas network in the present and future

We used the conservation planning software MARXAN (Ball & Possingham, 2000) to select protected areas network that meet different conservation targets for each endemic bird species while minimizing conflicts in denser populated counties. MARXAN’s conservation planning formulation is represented as follows:

minimize

\begin{eqnarray*}\sum _{i=1}^{m}{c}_{i}{x}_{i}+b\sum _{i1=1}^{m}\sum _{i2=1}^{m}{x}_{i1} \left( 1-{x}_{i2} \right) c{v}_{i1,i2} \end{eqnarray*}

subject to the constraint that all the representation targets are met

\begin{eqnarray*}\sum _{i=1}^{m}{a}_{ij}{x}_{i}\geq {t}_{j}{\forall }_{j} \end{eqnarray*}

where *m* is the total number of planning units, *c*_*i*_ is the cost of planning unit _*i*_, *x*_*i*_ is the binary decision variable indicating whether planning unit _*i*_ is selected (*x*_*i*_ = 1) or not (*x*_*i*_ = 0), and a_*ij*_ is the contribution of planning unit _*i*_ to target _*j*_. The remaining part of the mathematical problem in equation 1 is the “boundary length modifier” (BLM), which controls the aggregation of planning units (Ball & Possingham, 2000). Specifically, cv_*i*__1__,*i*__2_ represents a penalty for selecting planning unit _*I*_ but not selecting neighboring planning unit _*j*_. In equation 2, *t*_*j*_ is the amount of each target _*j*_ that must be selected.

We used the Atlantic Forest counties as the unity of analysis (i.e., planning units) as they are the smallest geopolitical unit considered for top-down decision-making in Brazil (Pinto & Grelle, 2011). We built a species’ presence-absence matrix per county under current and future conditions based on species’ environmentally suitable area. We included different conservation targets (i.e., a percentage of species’ environmentally suitable area to be protected) for each species based on species’ sensitivity to habitat disturbance, conservation status and size of environmentally suitable area. We used Parker, Stotz & Fitzpatrick (1996)’s classification of species sensitivity to disturbance, targeting to include at least 30% of the environmentally suitable area for species with “high” sensitivity, 15% for species with “medium” sensitivity and 0% for species with “low” sensitivity. At the same time, we used the classification of the International Union for Nature Conservation Red List (http://www.iucnredlist.org/), targeting to include at least 30% of the environmentally suitable area for species for “critically endangered”, 20% for “endangered” species, 10% for “vulnerable” and 0% for “least concern” or “near threatened” species. Finally, we also established targets based on the size of environmentally suitable area, based on IUCN (2001)’s criteria for threat using the size of species’ area of occupancy as guidance, therefore targeting to include at least 30% species with environmentally suitable area <10 km^2^, 20% for species with environmentally suitable area <500 km^2^, 10% for species with <2.000 km^2^ and 0% for species with >2.000 km^2^. Thus, species with higher sensitivity, threat and/or little environmentally suitable area received higher representation targets. We performed a sensitivity analyses, halving or increasing twofold these values, to evaluate the robustness of our results.

We used human population density per county as a surrogate for socio-economic development in our cost data. Thus, we considered counties with denser populations a lesser priority in selecting areas for the establishment of protected areas. For the future scenario, we assumed that the human population density among counties will maintain the same relative ranking, i.e., the counties that currently have the lowest population densities might increase population in the future, but so will other counties and, therefore, will continue having the lowest densities in the future. We used the 2013 estimates of human population size per county from the Brazilian Institute of Geography and Statistics (http://www.ibge.gov.br).

We run MARXAN 100 times with 10 million iterations for each climatic conditions (current and future), planning both ignoring and considering the use of the “boundary length modifier” (BLM). Planning ignoring BLM is the standard procedure, where counties are selected independently, providing county level solutions that are readily applicable at the smallest administrative unit for decision making in Brazil (hereafter, called “county level strategy”). The planning considering BLM aggregates counties in the final solution, and therefore, provides regional level solutions that are applicable at the state or federal administrative spheres (hereafter, called regional level strategy). We found the best BLM for each scenario by plotting total cost versus total edge of selected planning units (BLM) for the best solutions, and identifying BLM values where total cost and total edge intersects. We did so by running MARXAN with different BLM values, from 0 to 1 in installments of 0.1 (see Fig. S1). Furthermore, because a representative protected area network should be designed to build on top of the already existing network (Margules & Pressey, 2000), we required final solutions to always include the 15 counties that had >50% of their area already under protection (see Table S2 for the list of counties and their attributes), including only protected areas that can be considered as I-IV category in the IUCN, i.e., Brazilian strictly protected areas and Private Natural Heritage Reserve (Crouzeilles et al., 2013a). Spatial data on Brazilian protected areas was downloaded the Brazilian Ministry of Environment (http://www.icmbio.gov.br/portal/geoprocessamentos/51-m`enu-servicos/4004-downloads-mapa-tematico-e-dados-geoestatisticos-das-uc-s).

We calculated the percentage of overlap between the selected planning units in the most cost-effective solution under current and future conditions, planning both county and regional level strategies, in order to quantify the differences in the configuration between these protected area networks. Additionally, we calculated, for each species, the amount of environmentally suitable area protected under the solution of both scenarios (i.e., the overlapped of the most cost-effective solutions). All maps were processed in geographic coordinate system with Datum WGS84 and then converted to the World Cylindrical Equal projection. All analyses were carried out in ArcGIS 10.4 or R 2.12 (R Development Core Team, 2010).

### Prioritizing counties for protected area creation under climate change

An ad hoc prioritization scheme was created for counties that were selected in the most cost-effective solution under both current and future scenarios. The scheme places higher priority on the counties that were particularly relevant for the final proposed protected area network, had the greatest amount of forest remnant, and lowest area already within protected areas. A Priority Score was created, therefore, by summing the forest remnants (%) within the county + area outside protected areas (%) within the county + county’s irreplacebility under current conditions (%) + county’s irreplacebility under future conditions (%), where the irreplacebility is the number of times the county was selected in the 100 MARXAN runs. The maximum possible Priority Score, therefore, is 400 (i.e., 100% forest cover, 100% unprotected area, 100% irreplacebility under current condition and 100% irreplacebility under future conditions).

## Results

### Ecological niche modeling

**Figure 1:**
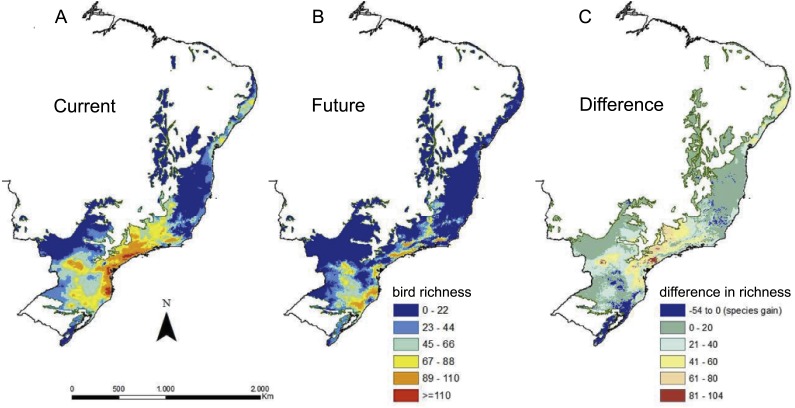
Richness of Atlantic Forest endemic birds based on ecological niche models. (A) Species’ richness under current conditions (max. = 116 spp.). (B) Species’ richness under future climate change conditions (max. = 110 spp.). (C) Difference in between richness under current and future conditions showing areas that lose (positive numbers) and areas that gain (negative numbers) species in the future.

Ecological niche models had, in general, a good performance, with BIOCLIM as the algorithm with the best results and MAXENT and GLM with the worst (Table 1). Most species (120 spp., 80%) had models with TSS greater than the minimum threshold in all algorithms, except for three species (*Touit melanonotus*, *Myrmotherula urosticta* and *Phylloscartes beckeri*), who’s consensus models had to be built from only two algorithms, because the other models were discarded for having poor performance measures. The models predicted a contraction of environmentally suitable areas under 2050 climate change conditions for the vast majority of the 151 species modeled (Fig. 1). For 148 species, we predicted an average contraction ∼29,500 km^2^ in environmentally suitable areas, representing 52% of the current area. Some species had particularly worrisome predictions, such as *Amazona brasiliensis*, with <50 km^2^ of environmentally suitable area by 2050 (99.1% area loss), or *Myrmotherula urosticta* and *Leptodon forbesi* with only 2 km^2^ (99.7% and 99.9% area loss, respectively). We also predicted that *Touit melanonotus* will lose 100% of suitable areas, but this result must be interpreted with caution, as the species’ final ensemble model was built with only two algorithms. For three species (*Thripophaga macroura, Amazona rhodocorytha, Polioptila lactea*), however, models predict an expansion of environmentally suitable area, with an average increase of 4,100 km^2^ or 10% greater than the current area (see Table S1 for species’ current and future environmentally suitable area).

**Table 1:** Performance of ecological niche models. For 151 birds endemic to the Brazilian Atlantic Forest, as measured by True Skill Statistics, for the five modeling algorithms used. Only models with TSS > 0.4 contributed to the final ensemble model for the species.

	BIOCLIM	DOMAIN	GLM	MAXENT	SVM
Average TSS value (standard error±)	0.75 (0.10)	0.62 (0.10)	0.6 (0.16)	0.58 (0.17)	0.68 (0.15)
Number of species with TSS >0.4	151	148	133	131	145

### Planning protected areas network under current and future conditions

Most species achieved their specific conservation targets, both under current and future conditions, independent of the use of county or regional level strategy (Table 2). Only three species did not achieve their specific conservation target in a specific combination of climatic conditions or strategy level: *Acrobatornis fonsecai*, *Phylloscartes beckeri* and *Tangara fastuosa*.

**Table 2:** Number of counties and species represented in counties selected. For the proposed protected area network, under current and future climate change scenarios. Results using a county level strategy (ignoring the boundary length modifier - BLM) and regional level strategy (considering BLM). Overlap refers to the counties selected in both current and future scenarios, with the number of and the species represented in the future.

	**Current**		**Future**		**Overlap**	
	Counties	Species	Counties	Species	Counties	Species
County level	466	150	484	147	256	128
Regional level	463	150	553	147	284	126

**Figure 2:**
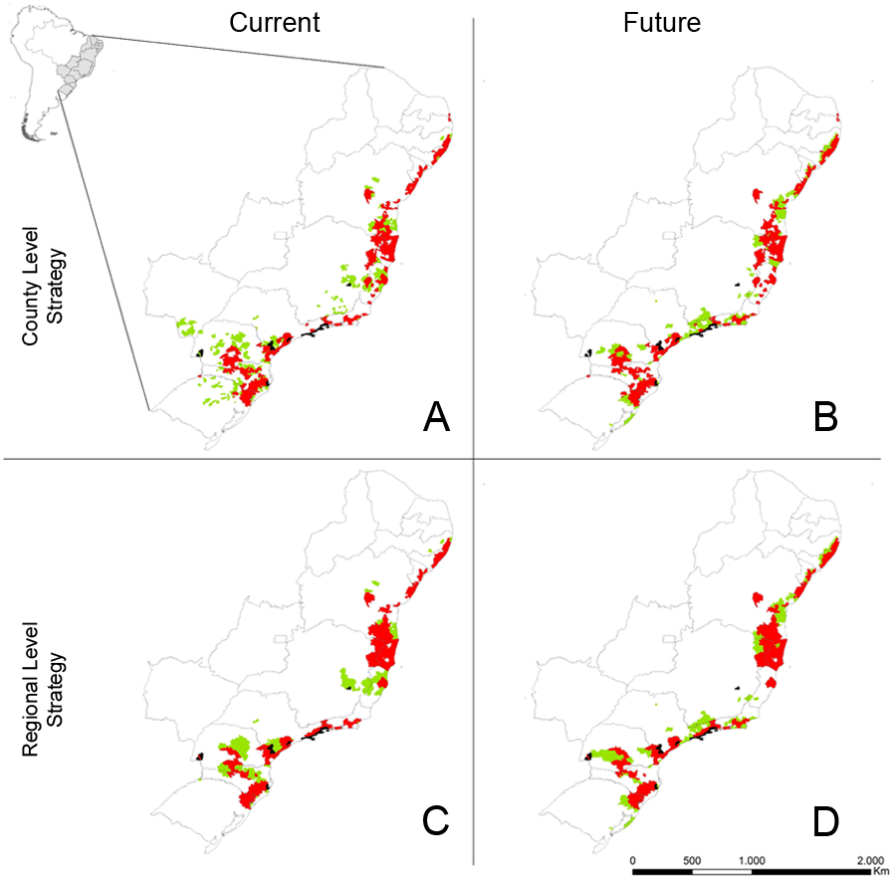
Selected counties for the establishment of protected areas networks for the conservation of Atlantic Forest endemic birds. We used a county level strategy (ignoring the boundary length modifier - BLM) and regional level strategy (considering BLM). Black counties already have protected areas and were always included in the final solution; red counties were selected in both current and future scenarios, while green counties were selected in either current or future scenario. (A) Selected counties using a county level strategy under current climatic conditions. (B) Selected counties using a county level strategy under future climatic conditions. (C) Selected counties using a regional level strategy under current climatic conditions. (D) Selected counties using a regional level strategy under future climatic conditions.

The final solutions (i.e., the most cost-effective) represented 150 and 147 of the 151 target species in the current and the future scenarios, respectively, independent of the use of county or regional level strategy (Table 2). Under current conditions, ca. 460 counties were required to achieve that representativeness, again with no relevant difference between county or regional level strategies (Table 2). Under future conditions, however, 484 and 553 counties were required to achieve that representativeness under the county and regional level strategies, respectively (Table 2). Such increase in the number of counties selected in the future for the regional level strategy resulted in a higher overlap of the most cost-effective solutions for current and future scenarios, from 256 (under the county level strategy) to 284. Notwithstanding, both county and regional level strategies, presented approximately 50% overlap in the most cost-effective solution for >83% of all species in current and future conditions (Fig. 2). We concluded, therefore, that for Atlantic Forest birds, there is a high congruence in protected areas network when planned under current and future conditions.

**Figure 3:**
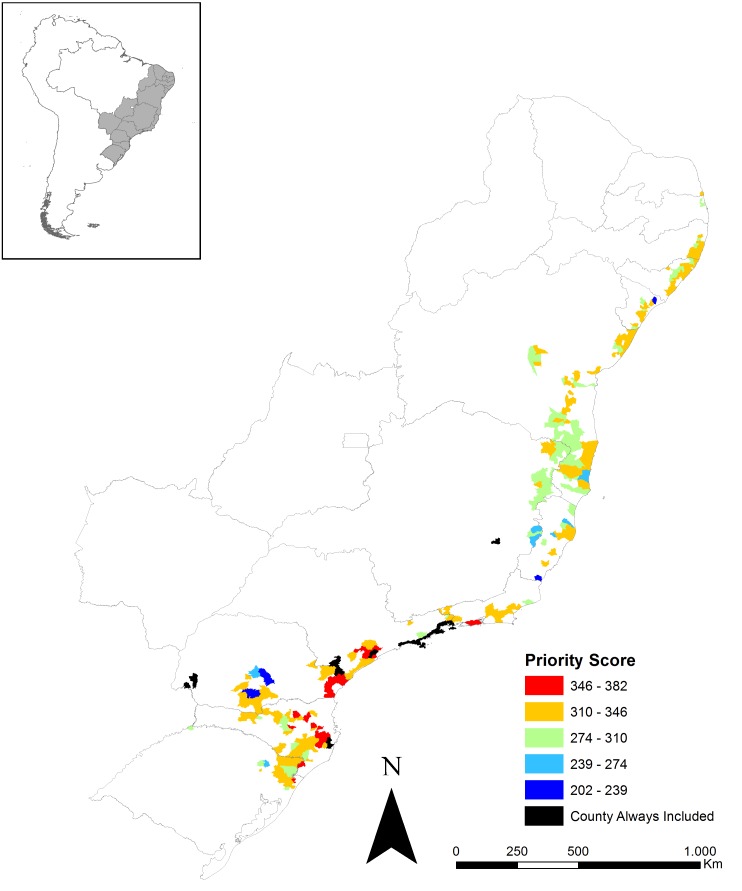
Counties’ priority score for protected area creation under climate change. Only counties that were selected both under current and future conditions are shown. Higher priority is placed on counties that were particularly relevant for the final proposed protected area network, had the greatest amount of forest remnant, and lowest area already within protected areas.

The Priority Score varied between 381 and 203 among counties selected both under current and future conditions, and highest scores prevailed among counties in the southern states of São Paulo, Paraná and Santa Catarina (Fig. 3, Table S2).

Although there is almost no numeric difference between county and regional level strategies, the spatial configuration of the two protected areas network was quite different (Fig. 2). As expected, the county level strategy produced a more fragmented selection of counties across the Atlantic Forest, while the regional level strategy produced a greater clumping of selected counties (Fig. 2).

The sensitivity analyses, halving or increasing twofold the conservation targets (i.e., a percentage of species’ environmentally suitable area to be protected), greatly decreased or increased the number of counties in the solution, respectively, but did not substantially change the number of species represented in the proposed protected area network (Fig. S2, Table S3). For the standard conservation strategy, for example, when halving the conservation target, there is an overlap of 126 counties between the solution under current and future conditions, representing 127 species. When doubling the conservation target, the number of counties needed to represent 137 species exploded to 1,629 overlapping counties, and almost the entire Atlantic Forest is selected (Fig. S2).

## Discussion

We found a large contraction in the environmentally suitable area for Atlantic Forest endemic birds under climate change scenarios, which is in agreement with previous studies in the biome with birds or other taxa (e.g., Souza et al., 2011; Lemes, Melo & Loyola, 2014; Loyola et al., 2014). The greatest losses in the future are predicted in the highly biodiverse central portion of the Atlantic Forest (Serra do Mar and Interior Forest subregions) (Jenkins et al., 2015), as well as in a more localized area in the north (Pernambuco sub-region (Fig. 1)), where only 1% to 7% of forest remnants are within protected areas (Ribeiro et al., 2009). At the same time, we predicted a moderate increase in species richness towards the south, as a result of shifting distribution (Fig. 1), which was also reported for other taxa (Colombo & Joly, 2010; Ferro et al, 2014; Lemes, Melo & Loyola, 2014). The general pattern of greater species richness in the central portion of the Atlantic Forest, however, persists under the future conditions (Fig. 1), which is one of the reasons why our study shows >50% overlap in selected counties under current and future conditions.

Another reason for the relatively large overlap in selected counties under current and future conditions is the use of counties as planning units. Most systematic conservation planning studies use regular units, such as squares or hexagons, which are typically smaller than Brazilian counties (e.g., Loyola et al., 2013; Crouzeilles et al., 2015 in the Atlantic Forest). The larger the planning unit, the greater the chance of overlap, but using counties as planning units is essential if the results are to be of any direct use for decision makers (Pinto & Grelle, 2011). This broad planning unit can also help to guide, with high return on investment, the allocation of limited resources for conservation. Our proposed protected areas network requires only ca. 10% of the Atlantic Forest counties for the most cost-effective solutions in both scenarios (overlap of current and future solutions). That is, the creation of new protected areas in these 10% of Atlantic Forest counties should be meaningful both right now and in the future.

Studies have shown that planning protected areas network under current environmental conditions tends to become less effective in species representation under future climatic change conditions (e.g., Hannah et al., 2007; Loyola et al., 2013). Our study shows a slightly more optimistic situation, with >50% overlap in selected counties and representation of >80% of the targeted species.

There are only two systematic conservation planning studies considering future climate change in the Atlantic Forest, despite the biome’s prominent biodiversity role (Loyola et al. (2013) with amphibians and Zwiener et al. (2017) with plants). Unfortunately, however, they do not provide results at the county level, hindering a comparison with our study and a direct guidance to policy makers. From an applied perspective, our recommendations for endemic birds in the Atlantic Forest are: (i) planning protected areas network currently can be useful to substantially represent species under climate change scenarios for 2050; (ii) counties that appear in the most cost-effective solution under current and future conditions can be considered “no regret” areas to invest in the establishment of protected areas; and (iii) decision can occurs at different administrative spheres as we found quite similar numerical solutions using either a county or regional level strategy.

Our proposed protected areas network using endemic birds as the target biological model has counties selected throughout the Atlantic Forest, even when using the regional level conservation strategy (Fig. 2). The availability of proprietary counties throughout the entire Atlantic Forest may be interesting, in practical terms, because it creates a flexible conservation portfolio. Conservation actions in Brazil are still often driven by local contingencies: a strong grass root movement in a certain area, a governor or mayor that is particularly sensitive to environmental issues, or the eligibility to a particular line of funding (e.g., Crouzeilles et al., 2015). Having priority counties spread throughout the biome, therefore, provides specific guidance wherever the opportunity of creating new protected areas arises.

Our results are intended to help decision makers by identifying priority counties in which conservation efforts should focus, i.e., those counties that combine high biodiversity with relatively low population density and, additionally, have relatively more area covered by forest and less covered by protected areas. After this first needed step, the specific location where each protected area should be created is contingent on other criteria and needs, such as landscape connectivity (e.g., Crouzeilles, Lorini & Grelle, 2013b). The administrative level in which decision occurs (county, state or federal) may directly affect the effectiveness of protected areas network. Our networks were quite similar numerically at either county or regional level strategies, meaning that our results are useful at different administrative spheres. In the state of Rio de Janeiro, for example, the Brazilian Ministry of the Environment (MMA in Portuguese acronym) and the State Institute of the Environment (INEA/RJ in Portuguese acronym) have encouraged the creation of new protected areas under the concept of “protected areas mosaic”, i.e., juxtaposed protected areas, with the same or different management regimes, that should be managed as a single unit (Crouzeilles, Lorini & Grelle, 2013b). The results of our regional level strategy provide clear guidelines for the establishment of new protected areas under the “mosaic” concept. Most states within the Atlantic Forest, however, have yet to join the mosaic concept, and for those, the results of our county level strategy can be more relevant.

Our study provides practical guidance on where to establish new protected areas in order to better protect Atlantic Forest birds against ongoing climate change. However, in biomes that have lost most of their original forest cover (such as the Atlantic Forest), the strategy of conserving the remaining forest under protected areas is often insufficient to safeguard biodiversity, precisely because of the scarcity of forest left. In such cases the protected area strategy must be complemented by restoration efforts in order to increase total available habitat and habitat connectivity, which are essential for species’ persistence under climate change conditions (Travis, 2003). Our proposed protected area network adequately represented >80% of the endemic birds of the Atlantic Forest, but the remaining 20% will likely require conservation strategies outside protected areas, such as forest restoration, in order to persist in the long-term. The enforcement of existing legislation represents a great opportunity for forest restoration. In the Atlantic Forest, the Brazilian Forest Code (Law no. 12.651) requires landowners to maintain a forest buffer of at last 30 m along rivers, and large landowners to maintain at least 20% of their property’s area as forest. The fact that only ca. 12% of the Atlantic Forest is actually forested (Ribeiro et al., 2009) demonstrates that most landowners do not comply with the law. The enforcement of the Forest Code could, therefore, be an important trigger for forest restoration in the Atlantic Forest. Still, there are many restoration initiatives in the Atlantic Forest outside the scope of Forest Code enforcement. The “Pact for the Restoration of the Atlantic Forest”, for example, congregates hundreds of independent restoration projects within the Atlantic Forest, with the goal of restoring 15 million hectares of forest by 2020 (http://www.pactomataatlantica.org.br). Currently, however, there is no study identifying priority areas for restoration within the Atlantic Forest. There is a clear need, therefore, for prioritization schemes for restoration in the Atlantic Forest that take into account not only conventional socio-economic and environmental constraints, but also the restoration potential for biodiversity conservation under future climate conditions.

## Conclusion

Protecting species today and then wait for climate change to unfold to improve the existing protected areas network is more expensive than planning both networks together (Hannah et al., 2007). Hence, identifying the congruencies between planning protected areas network under current and futures conditions, as we have done here, means to anticipate the impacts of climate change on biodiversity and provide more cost-effective solutions for its conservation. Currently, the network of protected areas within the Atlantic Forest does not adequately protect its endemic birds, because only a handful of the counties selected in the planned protected areas network currently have a protected area (Fig. 2). Here we provide a flexible portfolio of priority counties for decision makers. The establishment of new protected areas in the counties selected in this study may greatly improve the effectiveness of the current network of protected areas within the Atlantic Forest, both right now and in under future climate change conditions. However, because the Atlantic Forest have lost most of its forest cover, a similar analysis should be done focusing on prioritizing areas for forest restoration, in order to achieve a comprehensive strategy for biodiversity conservation in the Atlantic Forest under climate change.

##  Supplemental Information

10.7717/peerj.4689/supp-1 Figure S1 Best BLM for current and future scenarios Determined by plotting total cost (population density) versus total edge (BLM) for the best solutions, and identifying BLM values where total cost and total edge intersects. We did so by running MARXAN with different BLM values, from 0 to 1 in installments of 0.1.

10.7717/peerj.4689/supp-2 Figure S2 Results of the sensitivity analysis Halving or increasing 2-folds the conservation targets (i.e. a percentage of species’ environmentally suitable area to be protected). Selected counties for the establishment of protected areas networks for the conservation of Atlantic Forest endemic birds, under current and future climate change scenarios, using a county level strategy (ignoring the boundary length modifier - BLM) and regional level strategy (considering BLM).

10.7717/peerj.4689/supp-3 Table S1 Results per species Results per species showing species’ conservation status, number of occurrence records, results of the ecological niche modeling and systematic conservation planning analysis.

10.7717/peerj.4689/supp-4 Table S2 Results per county Raw data for each county in the study, including name, state, area (km^2^), population size, percentage forest remnants in 2011, percentage area within protected areas, current irreplaceability, future irreplaceability and summary result.

10.7717/peerj.4689/supp-5 Table S3 Results of the sensitivity analysis Halving or increasing 2-folds the conservation targets (i.e., a percentage of species’ environmentally suitable area to be protected). Number of counties and species represented in counties selected for the proposed protected area network, under current and future climate change scenarios. Results for the best solution using a county level strategy (ignoring the boundary length modifier - BLM) and regional level strategy (considering BLM).

## Additional Information and Declarations

### Data Availability

The following information was supplied regarding data availability: Data package title: Data from: Planning protected areas network that are relevant today and under future climate change is possible: the case of Atlantic Forest endemic birds DOI: http://dx.doi.org/10.5061/dryad.6n89r74.
