# Supplementary figures and images for: Planning protected areas network that are relevant today and under future climate change is possible: the case of Atlantic Forest endemic birds
Source: PeerJ. 2018 May 24;6:e4689. doi: 10.7717/peerj.4689 (PMC5971100; doi:10.7717/peerj.4689)

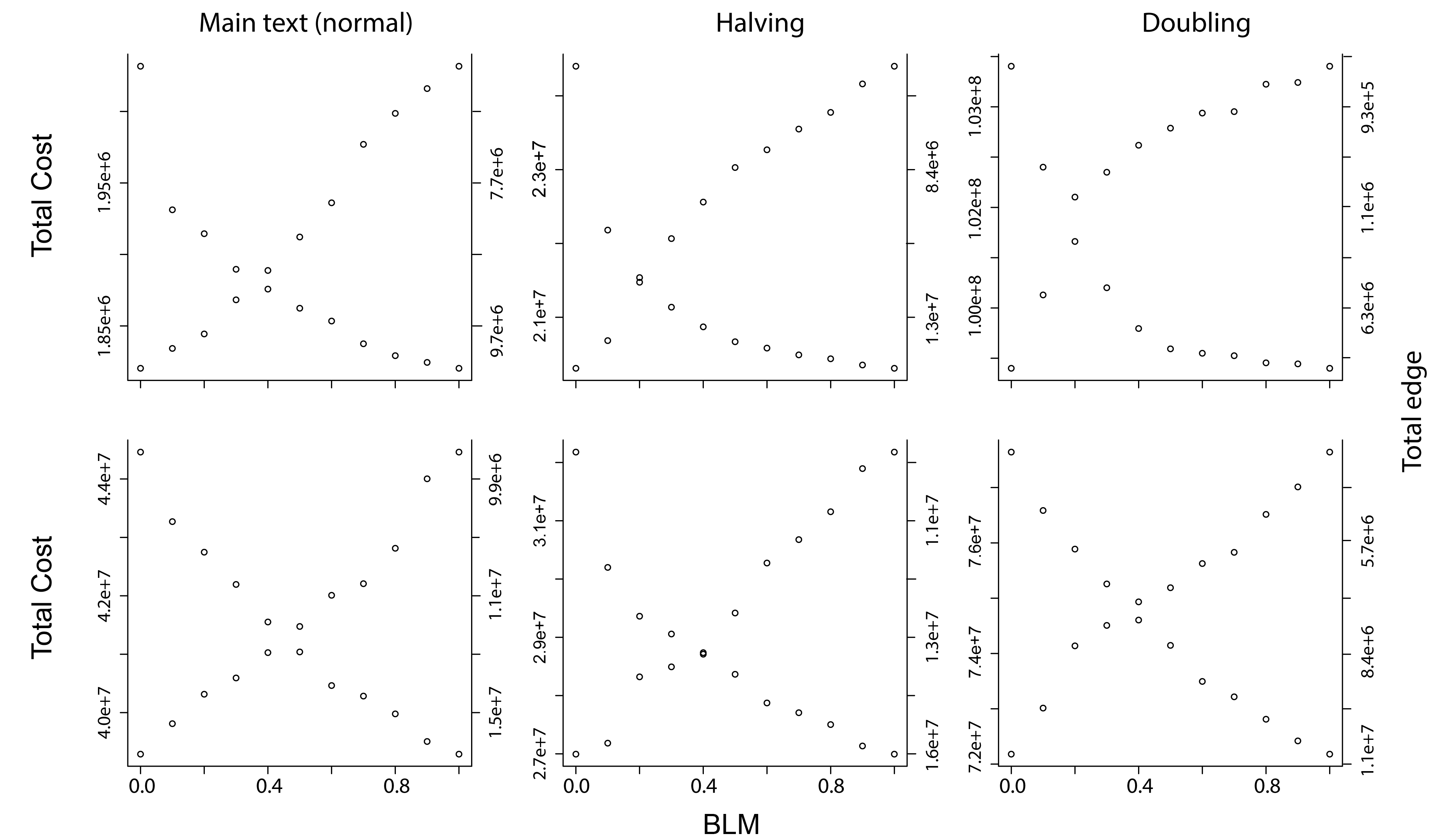

Supplement: Figure S1 — Determined by plotting total cost (population density) versus total edge (BLM) for the best solutions, and identifying BLM values where total cost and total edge intersects. We did so by running MARXAN with different BLM values, from 0 to 1 in installments of 0.1. [file peerj-06-4689-s001.png]

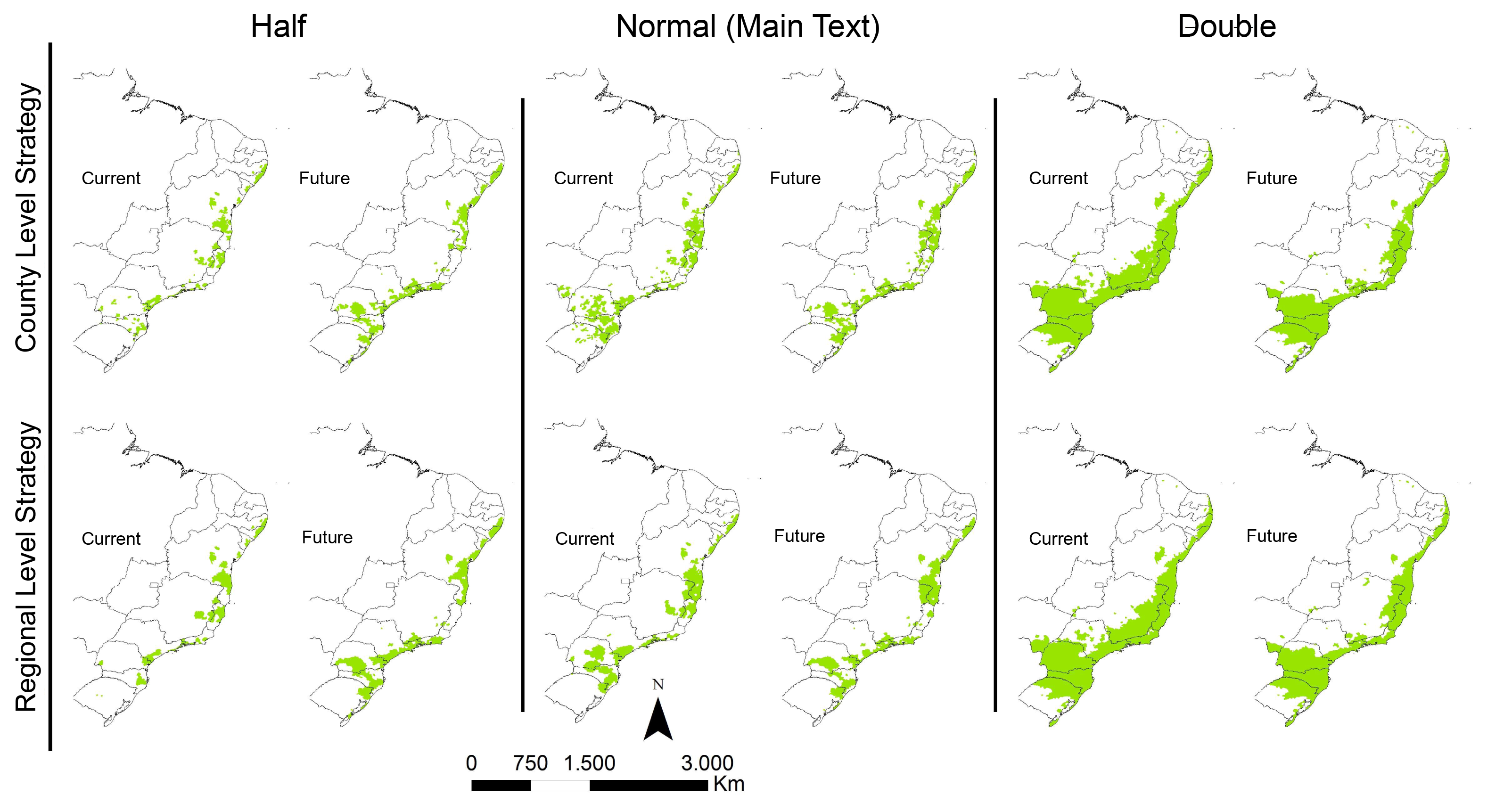

Supplement: Figure S2 — Halving or increasing 2-folds the conservation targets (i.e. a percentage of species’ environmentally suitable area to be protected). Selected counties for the establishment of protected areas networks for the conservation of Atlantic Forest endemic birds, under current and future climate change scenarios, using a county level strategy (ignoring the boundary length modifier - BLM) and regional level strategy (considering BLM). [file peerj-06-4689-s002.png]
