# Supplementary material for: Planning protected areas network that are relevant today and under future climate change is possible: the case of Atlantic Forest endemic birds
Source: PeerJ. 2018 May 24;6:e4689. doi: 10.7717/peerj.4689 (PMC5971100; doi:10.7717/peerj.4689)
Supplement: Table S3 — Halving or increasing 2-folds the conservation targets (i.e., a percentage of species’ environmentally suitable area to be protected). Number of counties and species represented in counties selected for the proposed protected area network, under current and future climate change scenarios. Results for the best solution using a county level strategy (ignoring the boundary length modifier - BLM) and regional level strategy (considering BLM). [file peerj-06-4689-s005.docx]

**Table S3**

|  |  | **Current** | | **Future** | | **Overlap** | |
| --- | --- | --- | --- | --- | --- | --- | --- |
| Strategy Level |  | counties | species | counties | species | counties | Species |
| County | **normal** | 466 | 150 | 484 | 147 | 256 | 128 |
| Regional |  | 463 | 150 | 553 | 147 | 284 | 126 |
| County | **halved** | 233 | 150 | 427 | 147 | 126 | 127 |
| Regional |  | 279 | 150 | 549 | 147 | 181 | 128 |
| County | **doubled** | 2,389 | 149 | 1,639 | 147 | 1,629 | 137 |
| Regional |  | 2,451 | 149 | 1,721 | 147 | 1,711 | 137 |
